# Hospital Almoners and Saturday Fund Subscribers

**Published:** 1913-02-15

**Authors:** Edgar S. Kemp

**Affiliations:** Hospital Almoners' Council, Denison House, Vauxhall Bridge Road, S.W.


*To the Editor of* The Hospital.

### Hospital Almoners and Saturday Fund Subscribers.

*To the Editor of* The Hospital.

Sir,—I have noted in your issue of February 8 the letter of Mr. Lewcock, of the Hospital Saturday Fund, drawing attention to the way in which subscribers to the Fund "are questioned and insulted by the so-called lady almoner at certain hospitals." Mr. Lewcock has made a serious charge against the almoners at certain hospitals, and it remains for him to substantiate it. He will, no doubt, be ready to inform your readers how many complaints he has received, who were the almoners implicated, and what were the results of the representations made on the patients' behalf to the hospitals concerned. I am assuming that these complaints have been brought to the notice of the hospital authorities.

In your editorial comment upon his letter you suggest that the statement deserves notice from the Hospital Almoners' Council. I would remind you that, as Secretary to the Council, I have no right to answer for the almoners at the hospitals, who, on appointment, become responsible only to the hospitals they serve; but, in view of the fact that the Council is vitally interested in the development of this valuable work, such adverse criticism is certainly of importance to it. Apart from this, there are a certain number of almoners working in the hospitals who have not been trained by my Council, and have no connection with it.

I note that, according to the *Hospital Saturday Fund Journal* of December 31, 1912, the Distribution Committee of the Hospital Saturday Fund has adopted a report recommending the general adoption of the almoner system.—Yours faithfully, Edgar S. Kemp.

Hospital Almoners' Council, Denison House, Vauxhall Bridge Road, S.W., February 11.

### The Hospital Saturday Fund.

